# Identical Twins with Leucine Rich Repeat Kinase Type 2 Mutations Discordant for Parkinson's Disease

**DOI:** 10.1002/mds.24924

**Published:** 2012-04-04

**Authors:** Georgia Xiromerisiou, Henry Houlden, Anna Sailer, Laura Silveira-Moriyama, John Hardy, Andrew J Lees

**Affiliations:** Reta Lila Weston Institute of Neurological Studies and Research Laboratories, Departments of Molecular Neuroscience and of Clinical Neuroscience, University College London (UCL) Institute of NeurologyQueen Square, London, UK

Analysis of concordancy rates in monozygotic and dizygotic twins with Parkinson's disease (PD) has been an important subject for research into the disorder^1^ and discordancy between twins has traditionally been interpreted as evidence against a genetic etiology of disease. Discordancy in late-onset diseases such as PD is complicated by the possibility that the disease onset may vary considerably between twins, and cases with up to 20 years of discordance have been reported.^2^ Leucine-rich repeat-kinase type 2 (LRRK2) mutations are the most common Mendelian cause of PD,^3^, ^4^ with the G2109S mutation occurring in 1% to 2% of idiopathic cases in the UK.^5^ Here we report the identification of a pair of identical twins with this mutation who are discordant by more than 10 years.

The twins, of English descent, are 70+ years old and were self-reported as identical. The proband developed the first symptoms of PD at age 60 years, with unilateral bradykinesia, rigidity, and rest tremor that became bilateral. The initial good response to levodopa therapy was followed in 5 years by development of motor fluctuations with wearing off, on-off effects, and peak dose and diphasic dyskinesias. The family had autosomal dominant inheritance of PD with a parent and 2 second-degree relatives affected by the disorder. On exam, the twin had a normal smell test and no signs of neurodegenerative disorder.

DNA from the proband was sequenced as part of the clinical workup and the heterozygous LRRK2 G2109S mutation was identified. DNA from the twin was sequenced and the mutation was confirmed in the sample. DNA from both twins was run on genomewide arrays (Illumina 660) to confirm that the twins were identical; this also revealed no major chromosomal abnormalities in either twin.

These data show that considerable variance in the penetrance of the mutation can occur even in the context of genetic identity. This suggests that the effects of other genetic loci in modifying the age at onset of disease must be minimal and, therefore, that identifying such loci through linkage or association methods will be extremely challenging because the variability in onset age between LRRK2 mutation carriers^6^ must be largely nongenetic in etiology. Identifying environmental risk factors for disease is notoriously difficult and there is nothing in the personal or medical histories of these twins that provides obvious clues for the reasons behind the current discordance. Indeed, both twins have had similar life courses. The recent data implicating pathology spread in PD is consistent with the notion that the disease process can start at a single site.^7^ If this is the case, then a stochastic initiation of disease may underlie the discordance as it may for prion disease.^8^, ^9^
